# Bacterial Species-Specific Activity of a Fluoroquinolone against Two Closely Related *Pasteurellaceae* with Similar MICs: Differential *In Vitro* Inoculum Effects and *In Vivo* Efficacies

**DOI:** 10.1371/journal.pone.0141441

**Published:** 2015-10-27

**Authors:** Guillaume Lhermie, Farid El Garch, Pierre-Louis Toutain, Aude A. Ferran, Alain Bousquet-Mélou

**Affiliations:** 1 Vétoquinol, Lure, France; 2 INRA, UMR1331 TOXALIM, Toulouse, France; 3 Université de Toulouse, INPT, ENVT, EIP, UPS, Toulouse, France; Second University of Naples, ITALY

## Abstract

We investigated the antimicrobial activity of a fluoroquinolone against two genetically close bacterial species belonging to the *Pasteurellaceae* family. Time-kill experiments were used to measure the *in vitro* activity of marbofloxacin against two strains of *Mannheimia haemolytica* and *Pasteurella multocida* with similar MICs. We observed that marbofloxacin was equally potent against 10^5^ CFU/mL inocula *M*. *haemolytica* and *P*. *multocida*. However, an inoculum effect was observed with *P*. *multocida*, meaning that marbofloxacin activity was decreased against a 10^8^ CFU/mL inoculum, whereas no inoculum effect was observed with *M*. *haemolytica*. Marbofloxacin activity was also tested in a lung infection model with immunocompromised mice intratracheally infected with 10^9^ CFU of each bacteria. At the same dose, the clinical and bacteriological outcomes were much better for mice infected with *M*. *haemolytica* than for those infected with *P*. *multocida*. Moreover, bacteriological eradication was obtained with a lower marbofloxacin dose for mice infected with *M*. *haemolytica*. Our results suggest that the differential *in vivo* marbofloxacin efficacy observed with the two bacterial species of similar MIC could be explained by a differential inoculum effect. Consequently, MICs determined on 10^5^ CFU inocula were not predictive of the differences in antibiotic efficacies against high bacterial inocula of closely related bacterial strains. These results could stimulate further investigations on bacterial species-specific antibiotic doses in a clinical setting.

## Introduction

In veterinary and human medicine, the fight against antimicrobial resistance requires the reduction of antimicrobials consumption through new regulatory requirements, the suppression of inappropriate uses, and also through the optimization of dosage regimens for appropriate uses. Such optimization of dosing regimens should more precisely take into account factors depending on the pathogens, the infection sites, the molecules, the hosts, and their interactions [1].

*Mannheimia haemolytica* and *Pasteurella multocida* are two Gram negative bacterial species belonging to the *Pasteurellaceae* family, which are closely genetically related. These bacteria are the most often encountered bacterial aetiological agents of a pulmonary disease in cattle called BRD complex. This disease can be treated by several antibiotics belonging to different families, including fluoroquinolones, and for a given antibiotic the same dosage regimens are registered for the overall disease independently of the bacterial aetiological agent. This situation of “disease claim” is representative of veterinary and human treatments targeting a cluster of pathogens. In the context of prudent use of antimicrobial drugs, we wondered if such “disease claim” could potentially be improved by more deeply exploring the activity of antibiotics against the different aetiological agents.

The efficacy of an antibiotic treatment depends among diverse factors on the susceptibility of the targeted pathogens, and it is the reason why the MICs of antibiotics used against bacteria are combined with pharmacokinetic parameters to explore the efficacy of dosage regimens. For fluoroquinolones, two PK/PD indices are correlated with the effectiveness of an antimicrobial treatment: AUC/MIC and Cmax/MIC [2, 3]. However, a set of studies have pointed out the limitations of MIC as the sole indicator to capture the complexity of interactions between bugs and drugs [4]. These limitations are linked to the standard procedure for its determination that consists in assessing antibiotic activity on an inoculum of 5.10^5^ CFU/mL in the presence of constant concentrations of drugs and after a fixed time of incubation [5]. As an example, the standard MIC determination does not take into account the inoculum effect whereas several studies have stressed that the activity of antibiotics is decreased when they face high bacterial inocula at the infection sites [6–10].

By using *in vitro* and *in vivo* assays, the aim of this study was to determine the activity of marbofloxacin, a fluoroquinolone used in veterinary medicine in Europe, against two strains of two closely related bacterial species belonging to the same Familiae (*Pasteurellaceae*) that have similar MICs and are targeted in the field by the same dosing regimens.

## Material and Methods

### Bacterial strains, media, antibiotic and reagents

A strain of *M*. *haemolytica* serotype *A2*, isolated in 2010 in the Netherlands from the lungs of a calf with clinical signs of BRD, and a strain of *P*. *multocida* isolated in a diseased pig in France were used in this study.

Mueller Hinton (MH) broth and agar and Brain Heart Infusion Broth (BHIB) were purchased from Biomerieux.

Marbofloxacin was provided by Vetoquinol SA and used for MIC and MBC determinations and for time-kill experiments. For *in vitro* testing, the powder was diluted in sterile distilled water from a stock solution at 1 mg/mL stored at -20°C. For *in vivo* testing (mice challenge), a commercial formulation of marbofloxacin for veterinary use approved in France (Marbocyl 10% ^®^) was used immediately after dilution in sterile distilled water.

### In vitro testing

#### Determination of the Minimum Inhibitory Concentration (MIC) and the Minimum Bactericidal Concentration (MBC)

MIC were determined in triplicate according to the CLSI reference methods (5). MIC were also determined in Brain Heart Infusion Broth (BHIB) with bacterial inocula of 5.10^5^ CFU/mL, using the same microdilution method since BHIB gave better growth of both strains and was used for killing curves experiments.

Bactericidal effects were determined with bacterial inocula of 10^5^ CFU/mL and 10^8^ CFU/mL, from time kill experiments by subculturing in MH agar plates, where wells with no visible growth were observed. The minimum bactericidal concentration (MBC) was defined as the lowest marbofloxacin concentration giving a decrease of more than 3log10 CFU/mL (99.9%) at 24h.

#### Time kill experiments

After overnight growth on MH agar plates, inocula of *M*. *haemolytica* and *P*. *multocida* were prepared by transferring several colonies into fresh BHIB to obtain suspensions containing 10^5^ CFU/mL and 10^8^ CFU/mL. Suspensions were incubated for one hour at 37°C to reach exponential growth phase before adding marbofloxacin at different concentrations (0, 0.25, 0.5, 0.75, 1, 2, 4, 8, 16 and 32 fold the MIC). Aliquots of 500 μL were removed from bacterial suspensions at 0, 0.5, 1, 2, 4, 6, 8 and 24h after incubation at 37°C. They were centrifuged at 3000g for 10 minutes and pellets were then resuspended in 500 μL of 0.9% NaCl to eliminate the carryover of marbofloxacin. Ten μL of successive 10-fold dilutions were plated on MH agar. The colonies were counted after 24h of incubation at 37°C. The detection limit of colony counting was 100 CFU/mL.

*Mean Inoculum growth*: From each time-kill curve, we calculated the Area Under the Curve (AUC) of bacterial counts from time 0 to 24h. This AUC was divided by 24 hours to obtain an average inoculum size over the investigated time interval (24 hours) for each x tested marbofloxacin concentration [3]. The basal inoculum (I_BASAL_) was equal to the inoculum size before exposure to marbofloxacin. Mean inoculum growth (ΔI(x)) was then defined by Eq 1:

$$\Delta \text{I}\left(\text{x}\right){=\text{I}}_{\text{x}}{\text{-I}}_{\text{BASAL}}$$ (1)

where ΔI(x) is expressed in log CFU/mL, I_x_ (log CFU/mL) is the mean inoculum obtained after exposure to a given marbofloxacin concentration x and I_BASAL_ (log CFU/mL) is the inoculum size before exposure to marbofloxacin.

*Modelling of mean inoculum growth*: Mean inoculum growth was modelled as a function of marbofloxacin concentration, using a sigmoid dose-response effect model implemented in the WinNonlin software version 5.3 (Pharsight, Moutain View). We re-parameterized equations previously used elsewhere [11, 12] which are derived from a classical sigmoid E_max_ model, to obtain Eqs 2 and 3 allowing to directly calculate two parameters of interest, *i*.*e*. EC_-3log_ the antimicrobial concentration producing a reduction of 3log_10_ (99.9% reduction) of the basal inoculum of 10^5^ CFU/mL, and EC_-5log_ the antimicrobial concentration producing a reduction of 5log_10_ (99.999% reduction) of the basal inoculum of 10^8^ CFU/mL.

ΔI(x)=ΔIMAX-(ΔIMAX-ΔIMIN)×(xEC-3log)γ((-3-ΔIMIN)(ΔIMAX+3))+(xEC-3log)γ (2)

ΔI(x)=ΔIMAX-(ΔIMAX-ΔIMIN)×(xEC-5log)γ((-5-ΔIMIN)(ΔIMAX+5))+(xEC-5log)γ (3)

where ΔI(x) (log CFU/mL) is the mean inoculum growth for marbofloxacin concentration x, ΔI_MAX_ and ΔI_MIN_ (log CFU/mL) the maximal and minimal inoculum growth, obtained without antibiotic (ΔI_MAX_) or after exposure to the highest marbofloxacin concentration (ΔI_MIN_), x (μg/mL) the tested marbofloxacin concentration, EC_-3log_ (μg/mL) the antimicrobial concentration producing a reduction in the basal inoculum of 3 log_10_, *i*.*e*. ΔI(EC_-3log_) = -3, EC_-5log_ (μg/mL) the antimicrobial concentration producing a reduction in the basal inoculum of 5 log_10_, *i*.*e*. ΔI(EC_-5log_) = -5, and γ the sigmoid coefficient of the curve. These values of the mean inoculum changes (-3 log_10_ and -5 log_10_) were chosen to take into account the size of the initial inoculum (5 log_10_ and 8 log_10_). In both cases, the remaining inoculum (2 or 3 log_10_) can be considered as similar, and low enough to be eradicated *in vivo* by the host natural defences.

### In vivo testing

#### Animals

All animal procedures were carried out in accordance with international accepted standards of animal care under agreement number TOXCOM/0022/AF GL (Ethic committee: Comité d’éthique de Pharmacologie‐Toxicologie de Toulouse‐Midi Pyrénées, France #C2EA-86) for animal experimentation of French Ministry of Agriculture.

Eighty female Swiss mice weighing 20 to 30g (Charles River Laboratories, L’Arbresle, France) and aged from 8 to 12 weeks were conditioned in our laboratory at least for one week before experimentation. Animals were housed with a 12 hours day-night cycle in a temperature (22°C) controlled room in autoclavable type II L cages with filters on Souralit^®^ (hygiene animal bedding) and had water and food (Tecklad global diet 2014 Harlan^®^) access ad libitum.

#### Induction of neutropenia

Cyclophosphamide (Sigma-Aldrich, St Quentin Fallavier, France) was administered intraperitoneally to mice at doses of 150 mg/kg 4 days before inoculation, and 100 mg/kg one day before inoculation.

#### Lung infection model

Mice were purchased specific pathogen free and the health status was checked twice a day before and during experiments. We verified mobility, respiratory movements and piloerection. If immobility was observed, we euthanized animals with the humane method described below. These animals were included in the “dead group”. If piloerection and/or increase of respiratory movements were detected, the mice were more carefully observed during the day.

On day 0, mice were anesthetized by an intraperitoneal injection of a mixture of 0.17 mg/kg medetomidine (Domitor^®^, Zoetis, France) and 80 mg/kg ketamine (Chlorketam^®^, Vétoquinol, France).

The tracheas were cannulated with a 22 G catheter without mandrel. The lungs were inoculated with 20μL of *M*. *haemolytica* or *P*. *multocida* suspension containing 5x10^11^ CFU/mL, corresponding to a total of 1x10^9^ CFU/lung. To constitute a control, 2 mice remained uninfected and were isolated in a separate box.

Three hours after bacterial challenge, mice received a single intraperitoneal injection of marbofloxacin. Eight groups of 10 to 13 mice were randomly constituted and were treated with 0, 1, 5, 10, 20 or 40 mg/kg of marbofloxacin for the mice inoculated with *M*. *haemolytica*, and with 0 or 40 mg/kg of marbofloxacin for the mice inoculated with *P*. *multocida*. For ethical reasons, only 4 to 8 mice were allocated in groups with low variability.

Clinical signs of disease and mortality were recorded over 48h, and at day 2 after challenge, all surviving mice were sacrificed humanely by an intraperitoneal injection of 120 mg/kg pentobarbital sodium (Dolethal^®^, Vétoquinol, France). The lungs were aseptically removed and homogenized in 5mL of 0.9% NaCl. The homogenates were centrifuged at 3000 g for 10 minutes and the pellets resuspended in 2 mL of 0.9% NaCl. Ten μL of successive 10-fold dilutions were then plated on MH agar. The bacterial counts per lung were recorded after 24h of incubation at 37°C. The detection limit of colony counts was 200 CFU/lung. Mice were then divided into 3 categories regarding the effect of marbofloxacin: “persistent infection” (decrease in bacterial load <3log10 CFU in lungs), “bactericidal effect” (decrease in bacterial load >3log10 CFU in lungs), “bacterial eradication” (bacterial counts below the LOQ). In the follow-up, dead mice were classified into the “persistent infection” category.

#### Statistical methods

The bacterial counts in lungs after 48 h were compared with an analysis of variance followed by a Dunnett comparison.

## Results

### In vitro testing

#### MIC and MBC results

For both bacterial species the values of MIC, MBC and the MBC/MIC ratio are shown in Table 1. The MIC values of marbofloxacin were 0.03 μg/mL for *M*. *haemolytica*, and 0.016 μg/mL for *P*. *multocida*. These values were identical in MHB and BHIB.

**Table 1:** Marbofloxacin MIC and MBC determined in BHIB for *M*. *haemolytica* and *P*. *multocida*.

	MIC (μg/mL)	MBC (μg/mL)	MBC/MIC ratio
*M*. *haemolytica*	0.03		
low inoculum		0.023	0.75
high inoculum		0.03	1
*P*. *multocida*	0.016		
low inoculum		0.03	2
high inoculum		0.12	8

MIC were determined with a standard inoculum of 5.10^5^ CFU/mL.

MBC were extracted from time-kill experiments.

Low inoculum: 5.10^5^ CFU/mL. High inoculum: 5.10^8^ CFU/mL.

The MBC values observed with the standard inoculum of 5.10^5^ CFU/mL showed very slight differences, *i*.*e*. equal or less than one 2-fold dilution, compared to the corresponding MIC values for both *M*. *haemolytica* (0.023 μg/mL) and *P*. *multocida* (0.03 μg/mL), as indicated by MBC/MIC ratios (Low inoculum, Table 1). The MBC value obtained with the 5.10^8^ CFU/mL inoculum was equal to the MIC (0.03 μg/mL) for M. *haemolytica*, but was 8-fold the MIC (0.12 μg/mL) for *P*. *multocida* (High inoculum, Table 1).

#### Time-kill curves

**Fig 1:**
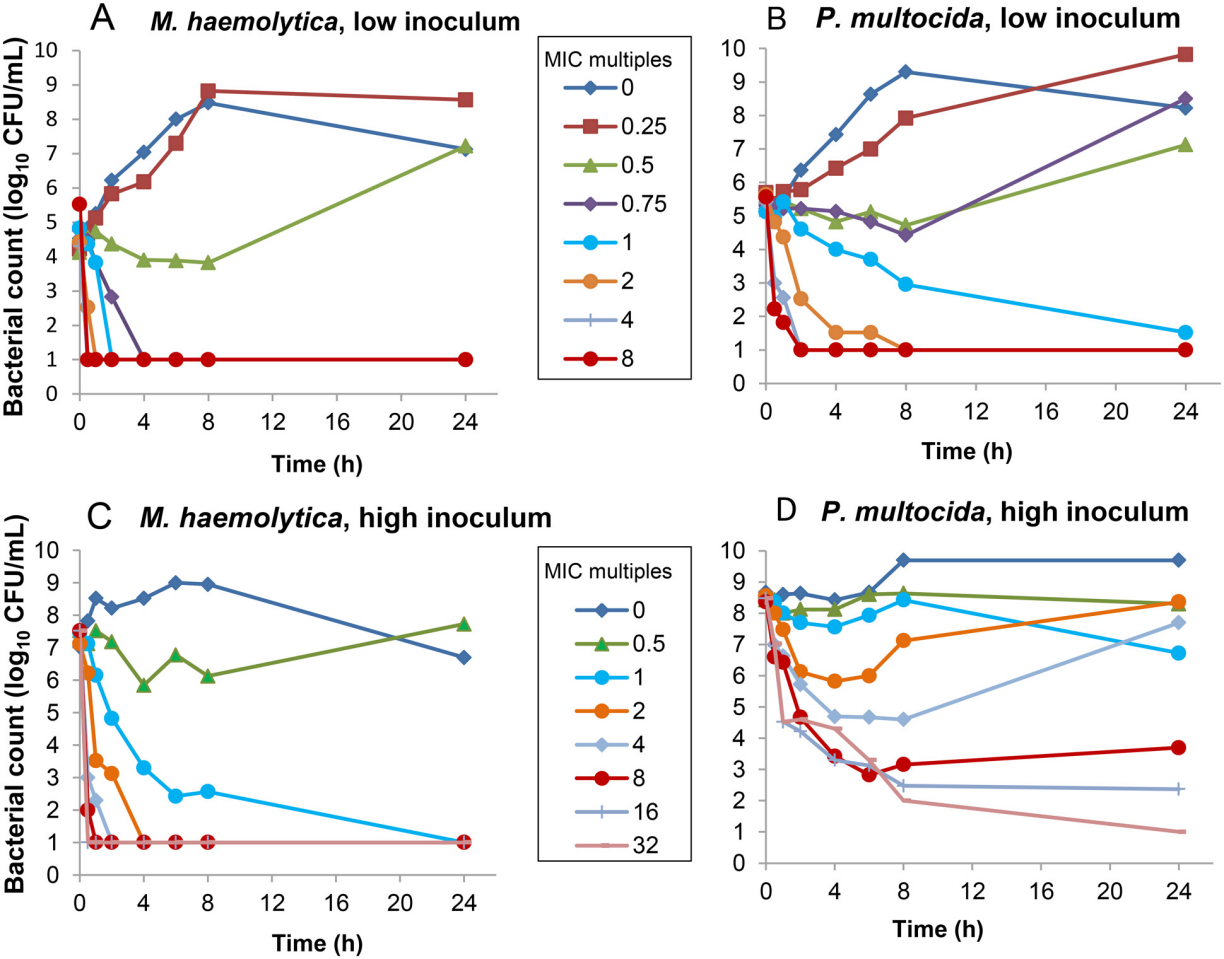
Time-kill curves of low (10^5^ CFU/mL) (A and B) and high (10^8^ CFU/mL) (C and D) inocula of *M*. *haemolytica* (A and C) and for *P*. *multocida* (B and D) in the presence of marbofloxacin (concentrations shown in multiple of the MIC (MIC = 0.016μg/mL for *P multocida* and MIC = 0.03 μg/mL for *M*. *haemolytica*).

The time-kill curves of *M*. *haemolytica* and *P*. *multocida* exposed to different concentrations of marbofloxacin are presented in Fig 1.

For *M*. *haemolytica*, we obtained a concentration-dependant bactericidal effect. For the low inoculum of 10^5^ CFU/mL the MBC was equal to 0.75xMIC. Marbofloxacin killed 99.9% of the bacteria within 0.5h at concentrations higher than 2xMIC, within 2h at 1 xMIC and within 4h at 0.75xMIC.

For the high inoculum (10^8^ CFU/mL), marbofloxacin killed 99.9% of the bacteria within 0.5h at concentrations ≥ 4xMIC, within 1h at 2xMIC, and within 2h at 1xMIC.

For *P*. *multocida*, the results obtained with the low inoculum of 10^5^ CFU/mL were similar to those observed with *M*. *haemolytica*. The MBC of marbofloxacin was equal to 1xMIC, even if the effect was slower than for *M*. *haemolytica* (killing <3log10 at 8h). In addition, marbofloxacin achieved 99.9% killing within 0.5, 1 and 2h at concentrations of 8x, 4x and 2xMIC, respectively. For the high inoculum of 10^8^ CFU/mL *P*. *multocida*, the MBC was ≥ 8xMIC; marbofloxacin achieved 99.9% killing within 1h at concentrations ≥ 16xMIC, 2h at 8xMIC. At 4xMIC, a transitory bactericidal activity was observed for the first 8 hours but a regrowth was observed at 24h.

#### Pharmacodynamic modelling

**Fig 2:**
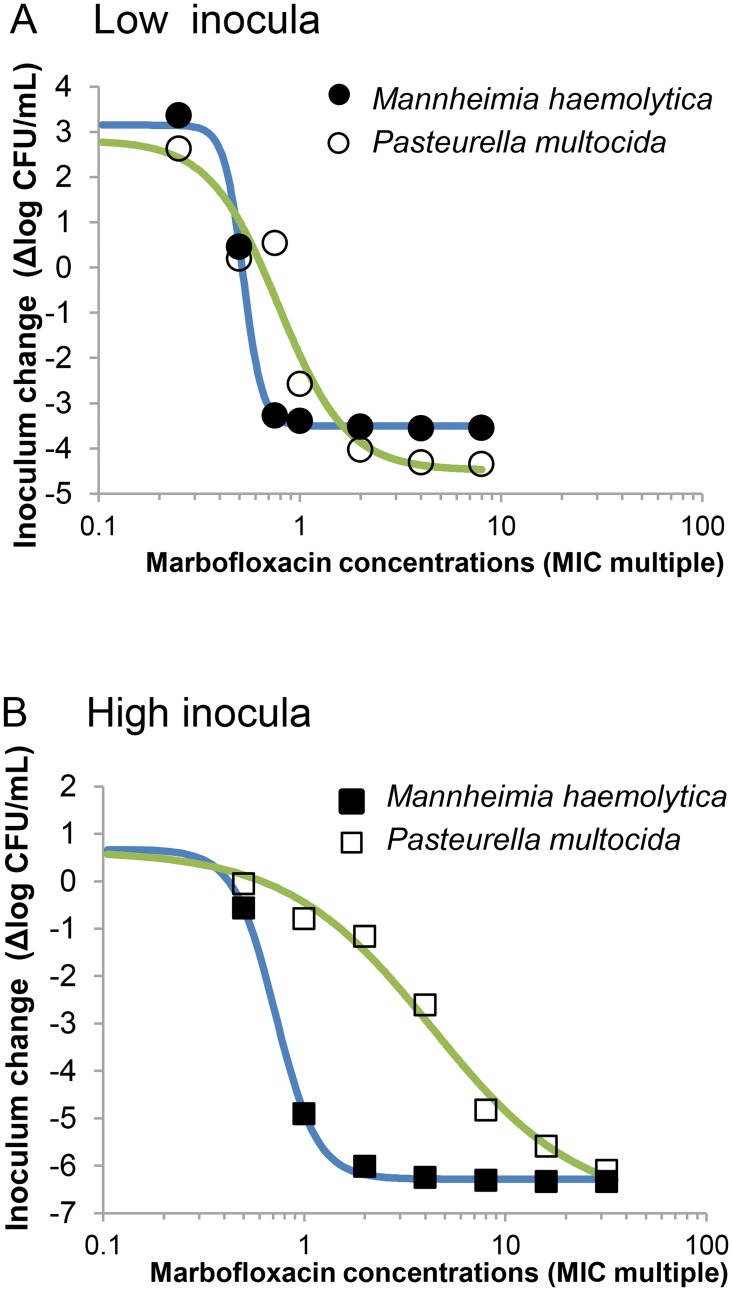
Bactericidal effect of marbofloxacin against low (A) and high (B) inocula of *M*. *haemolytica* (full symbols) and *P*. *multocida* (open symbols) isolates.

The relations between inoculum changes and marbofloxacin concentrations are presented in Fig 2. The observed data were fitted with the models described by Eqs 2 and 3 and the pharmacodynamic parameters are presented in Table 2.

**Table 2:** Pharmacodynamic parameters^a^ describing the antimicrobial activity of marbofloxacin against different inoculum sizes of *M*. *haemolytica* (MH) and *P*. *multocida* (PM) in BHIB.

Basal inoculum	Bacteria	EC_-3log_ ^b^ or EC_-5log_ ^c^	PM/MH	γ^d^	ΔI_MAX_ ^e^	ΔI_MIN_ ^f^
(Log_10_ CFU/mL)		(MIC multiple)	ratio		(Log_10_ CFU/mL)	(Log_10_ CFU/mL)
5	*MH*	0.69±0.05 ^b^		9.14±2.12	3.15±0.12	-3.50±0.09
	*PM*	1.34±0.29 ^b^	1.95	2.53±1.06	2.82±0.69	-4.49±0.60
8	*MH*	1.03±0.02^c^		4.17±0.21	0.67±0.10	-6.28±0.04
	*PM*	10.0±1.53^c^	9.71	1.34±0.32	0.57±0.35	-6.70±0.65

^a^ Mean ±SE of parameters calculated with Eqs 2 and 3 (sigmoid E_max_ model)

^*b*^ Antibiotic concentration reducing by 3 log_10_ the basal inoculum of 10^5^ CFU/mL

^*c*^Antibiotic concentration reducing by 5 log_10_ the basal inoculum of 10^8^ CFU/mL

^*d*^ Sigmoid coefficient of the curve.

^e^ Maximal growth compared to the basal inoculum.

^f^ Minimal growth (maximal killing) compared to the basal inoculum.

For the low inoculum of both *M*. *haemolytica* and *P*. *multocida*, concentration-effect curves were close, with marbofloxacin concentrations associated with ΔI = -3 around 1xMIC (see Fig 2, upper panel). Indeed, data fitting using Eq 2 indicated that EC_-3log_ (concentrations associated with ΔI = -3) were 0.69xMIC and 1.34xMIC for *M*. *haemolytica* and *P*. *multocida*, respectively. The ratio of EC_-3log_ for *P*. *multocida* and *M*. *haemolytica* was 1.95 (Table 2).

For the high inoculum, the concentration-effect curve of *M*. *haemolytica* (Fig 2, lower panel) showed that inoculum changes occurred in a concentration range of marbofloxacin similar to that observed with the low inoculum of *M*. *haemolytica* (between 0.5 and 2- fold the MIC). Conversely, the concentration-effect curve for the high inoculum of *P*. *multocida* was dramatically shifted to the right, indicating that the marbofloxacin concentrations required to achieve the same magnitude of inoculum change were much higher with *P*. *multocida* than with *M*. *haemolytica*. Data fitting using Eq 3 indicated that EC-_5log_ (concentrations associated with ΔI = -5) were 1.03xMIC and 10.0xMIC for *M*. *haemolytica* and *P*. *multocida*, respectively (Table 2). The ratio of EC_-5log_ for *P*. *multocida* and *M*. *haemolytica* was 9.74 (Table 2).

### Infection model

#### Clinical outcomes: mouse survival

Mice that were not infected showed normal activity and no death, during the whole experiment. The results obtained in the different groups of infected mice are presented in Table 3.

**Table 3:** Effect of the different marbofloxacin doses on the survival and clinical and bacteriological cure of mice infected with *P*. *multocida* or *M*. *haemolytica*.

				*M*. *haemolytica*			*P*. *multocida*	
Marbofloxacin dose (mg/kg)	0	1	5	10	20	40	0	40
Number of mice	13	4	10	12	12	8	4	11
% of mice with clinical signs	100	100	60	58	20	0	100	100
% of surviving mice	85	100	100	100	100	100	0	100
% of mice with bacterial eradication	0	0	10	25	33	37	0	0
% of mice with bactericidal effect	0	0	50	33	58	63	0	9
% of mice with persistent infection	100	100	40	42	9	0	0	9

Bacterial eradication: bacterial counts below the LOQ; bactericidal effect: decrease in bacterial load >3log10 CFU; persistent infection: decrease in bacterial load<3log10 CFU.

In the two infected/non-treated groups (0 mg/kg marbofloxacin dose), all the mice infected with *M*. *haemolytica* or *P*. *multocida* presented clinical signs of disease. About 15% (2/13) of the mice infected with *M*. *haemolytica* died between 36–48 h post-challenge, and 100% (4/4) of the mice infected with *P*. *multocida* died between 12–36 h post-challenge (85% and 0% survival at 48 h, respectively, Table 3).

In the group of mice infected with *P*. *multocida* and treated with the 40 mg/kg marbofloxacin dose, 100% (11/11) of the mice showed persistent clinical signs of infection (they developed mildly scruffy coat and were prostrate) and were still alive at 48h post-challenge (Table 3).

In the groups of mice infected with *M*. *haemolytica* and treated with the 40 mg/kg marbofloxacin dose, 100% survival was observed at 48 h post-challenge and none of the mice showed any clinical signs of disease. When using decreasing doses of marbofloxacin (20, 10, 5 and 1 mg/kg) in mice infected with *M*. *haemolytica*, 100% survival was observed at 48 h post-challenge and the percentage of mice presenting clinical signs progressively increased from 20% to 100% (Table 3).

#### Microbiological outcomes: pulmonary bacterial loads

**Fig 3:**
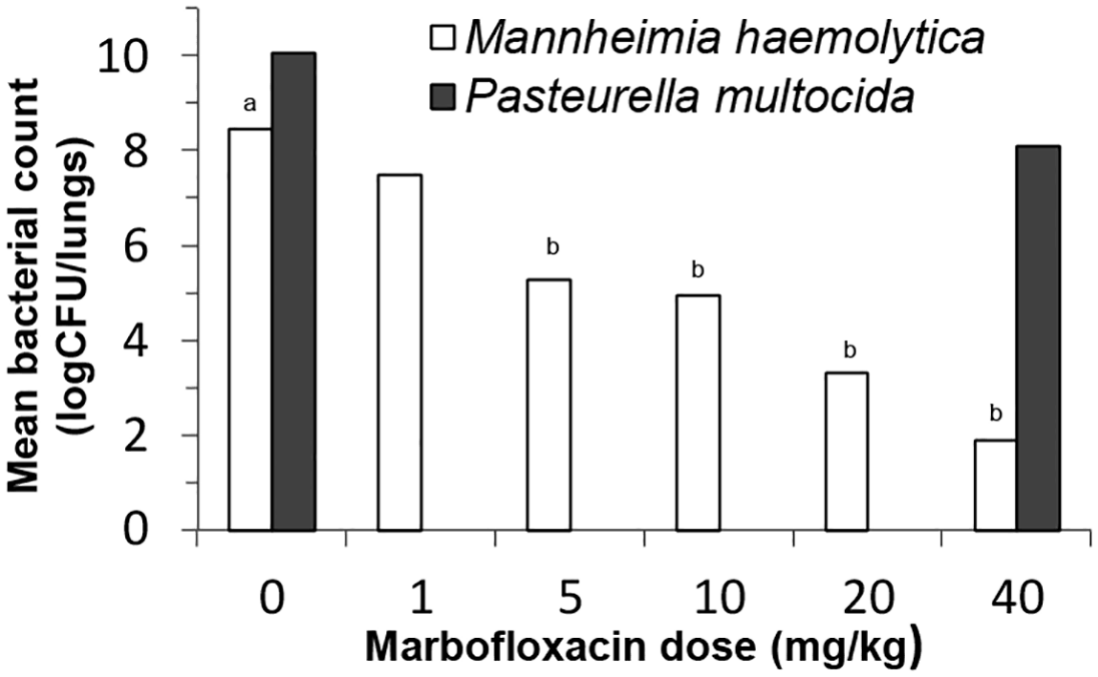
Mean bacterial counts in the lungs of mice infected with *P*. *multocida* (dark bars) or with *M*. *haemolytica* (white bars) after intraperitoneal administrations of different doses of marbofloxacin. Different letters in superscript above bars indicate that values are statistically different.

The lung bacterial counts measured in mice 48h after the challenge are reported in Fig 3.

The mean bacterial counts in the lungs of untreated animals infected with *M*. *haemolytica* and *P*. *multocida* were 8.48±0.97 and 10.08±0.06 log_10_ CFU/lung, respectively.

The 40 mg/kg marbofloxacin dose led to a dramatic decrease in the mean bacterial counts for *M*. *haemolytica* infected mice (1.91±0.90 log_10_ CFU/lung) compared to the untreated group (8.48±0.97 log_10_ CFU/lung), whereas the mean bacterial counts were only slightly decreased for *P*. *multocida* infected mice (8.10±1.30 *versus* 10.08±0.06 log_10_ CFU/lung). For *M*. *haemolytica* infected mice, decreasing the marbofloxacin doses from 20 to 1 mg/kg showed a progressive increase in the mean bacterial loads in the lungs at 48h post-challenge. An analysis of variance followed by a Dunnett comparison indicated that the mean bacterial count for 1 mg/kg was not significantly different (p>0.05) from the untreated group, whereas the mean bacterial counts were significantly decreased compared to the control group for the 5 mg/kg and higher doses (p<0.05).

The percentages of mice exhibiting “persistent infection” (decrease in bacterial load <3log10 CFU), “bactericidal effect” (decrease in bacterial load >3log10 CFU), or “bacterial eradication” (bacterial counts below the LOQ) are presented in Table 3.

The 40 mg/kg marbofloxacin dose produced bacterial eradication or a bactericidal effect in 37% and 63% of mice infected with *M*. *haemolytica*, respectively. In mice infected with *P*. *multocida*, the 40 mg/kg marbofloxacin dose produced no bacterial eradication, and only a 9% bactericidal effect.

In mice infected with *M*. *haemolytica*, the decrease in marbofloxacin doses from 20 to 1 mg/kg was correlated with an increase in the percentage of mice with persistent infection: 9%, 42%, 40% and 100% for the marbofloxacin doses of 20, 10, 5 and 1 mg/kg, respectively.

## Discussion

In the present study, we assessed in a mouse model of pulmonary infection the efficacy of an antimicrobial treatment with marbofloxacin against two bacterial species belonging to the *Pasteurellaceae* family: *M*. *haemolytica* and *P*. *multocida*. These two bacterial species are among the etiologic agents of the same disease in cattle.

Our main result demonstrated that the dose of marbofloxacin required to cure experimental pneumonia in mice was dramatically different depending on the bacterial pathogen responsible for the infection, despite the fact that these pathogens have similar MICs and are closely genetically related (*Pasteurellaceae* family). Moreover, *in vitro* assessment of the antibacterial activity of marbofloxacin against both bacterial species demonstrated that the inoculum effect, that corresponds to the decrease of antibacterial activity when inoculum size increases, and which was previously identified as an important factor of antimicrobial efficacy of fluoroquinolone [9, 13, 14], was also dramatically different in magnitude between the two bacterial species.

Our first objective was to develop a rodent model of pulmonary infection with *M*. *haemolytica* or *P*. *multocida* to enable clinical and bacteriological outcomes to be assessed after marbofloxacin treatments. In our experiments, we chose to depress the immune response of the mice, by the administration of cyclophosphamide before the course of challenges [15] to get similar bacterial counts at the time of treatment with both bacteria. By minimizing the implication of the natural (specific and non-specific) defence, neutropenia enabled overt disease to be established in order to compare the effects of marbofloxacin on both bacterial species.

In this pneumonia model, we first evaluated the clinical and microbiological outcomes after marbofloxacin treatment at a dose of 40 mg/kg. This dose had already been tested in a pneumonia model with *P*. *multocida* in immunocompetent mice [16]. In the present study with immunocompromised mice infected by *P*. *multocida*, the 40 mg/kg dose prevented mortality at 48 h post-challenge, but it produced neither a clinical cure nor a significant decrease in the bacterial loads in the lungs. Conversely, the 40 mg/kg dose in immunocompromised mice challenged with *M*. *haemolytica* was associated with a 100% clinical cure rate and a significant microbiological effect in all treated mice (including a microbiological cure rate of 40%). We then decided to explore the magnitude of this differential drug efficacy by treating *M*. *haemolytica* infected mice with decreasing doses of marbofloxacin. For ethical reasons, as the 40 mg/kg dose was poorly effective against *P*. *multocida*, we did not test lower doses of marbofloxacin in *P*. *multocida* infected mice. After testing marbofloxacin doses of 20, 10, 5 and 1 mg/kg, we demonstrated that the 5 mg/kg dose was the lowest dose producing better clinical and microbiological outcomes in the *M*. *haemolytica* pneumonia than the 40 mg/kg marbofloxacin dose in the *P*. *multocida* pneumonia (Fig 3 and Table 3).

We explored *in vitro* using time-kill experiments the antibacterial activity of marbofloxacin against both *P*. *multocida* and *M*. *haemolytica* strains. The bacteriological results were expressed in terms of mean inoculum changes over 24 h, and analysed as a function of marbofloxacin concentrations [11, 12]. With initial inocula of 10^5^ CFU/mL, the marbofloxacin concentrations producing a 99.9% (-3 log_10_) reduction in the basal inoculum were similar between the two bacterial strains and close to their respective MICs (0.69xMIC for *M*. *haemolytica* and 1.34xMIC for *P*. *multocida*, Table 2). Similar results were obtained by Ferran et al. [12] with *P*. *multocida* (2.06xMIC) and *E*. *coli* (1.80xMIC). This is in agreement with the observation that the MBC of marbofloxacin was close to the MIC for both *P*.*multocida* and *M*. *haemolytica*. Altogether, these results support the view that MIC is a relevant indicator of the marbofloxacin bactericidal effect against low bacterial inocula.

Using initial inocula of 10^8^ CFU/mL, huge differences were observed when comparing the marbofloxacin activity against *P*. *multocida* and *M*. *haemolytica* strains (Fig 2, lower panel; Table 2).

For *P*. *multocida*, the concentration-response curve was shifted to the right of the x-axis, and the marbofloxacin concentration that gave a 99.999% (-5 log_10_) reduction in the basal inoculum was about 10-fold the MIC. These results are evidence of the inoculum effect, consisting of a reduction in the antibiotic activity while bacterial inoculum size increases. This effect was previously demonstrated with *P*. *multocida* [12] and with other Gram-negative and Gram-positive bacterial species [9, 13, 14, 17].

Unexpectedly, the concentration-response curve for *M*. *haemolytica* remained close to that obtained with the 10^5^ CFU/mL inoculum, and the marbofloxacin concentrations associated with a 99.999% (-5 log_10_) reduction in the basal inoculum was 1.03xMIC which is similar to the value obtained with a -3 log_10_ reduction of the 10^5^ CFU/mL inoculum (0.69xMIC). The same result was obtained with another strain of *M*. *haemolytica* (data not shown). *M*. *haemolytica* is, to our knowledge, the first reported bacterial species that does not exhibit such an inoculum effect for a fluoroquinolone.

Interestingly, the results obtained *in vitro* with the high inocula (10^8^ CFU/mL) of *P*. *multocida* and *M*. *haemolytica* (Fig 2) mirror those obtained *in vivo* in the pneumonia model produced with a bacterial load of 10^9^ CFU/lung. Indeed, the ratio of *in vitro* effective concentrations (EC_-5log_) for *P*. *multocida* and *M*. *haemolytica* was equal to 9.71 (Table 2), and the ratio of *in vivo* doses producing similar responses for *P*. *multocida* and *M*. *haemolytica* was about 8 (40 mg/kg for *P*. *multocida* and 5 mg/kg *M*. *haemolytica*).

In a set of published works, it has been demonstrated in experimental models of infections that the antimicrobial activity of fluoroquinolones or third-generation cephalosporins could be reduced against high bacterial loads compared to low bacterial loads of different bacterial species, including *Escherichia coli*, *Pasteurella multocida*, *Klebsiella pneumoniae* and *Staphylococcus aureus* [6, 9, 13, 18]. In all these cases, *in vitro* time-kill experiments have shown the occurrence of an inoculum effect.

Altogether, these data suggest that the higher activity of marbofloxacin in the pneumonia induced by a high inoculum of *M*. *haemolytica* compared to *P*. *multocida* was potentially linked to the absence of an inoculum effect in *M*. *haemolytica*. Therefore, it might be speculated that marbofloxacin was as effective against the high inoculum of *M*. *haemolytica* as it would be against a low inoculum. Unfortunately, this situation could not be tested in our model of infection because of the low pathogenicity of *M*. *haemolytica* in mice, which did not produce overt clinical symptoms of disease with a low bacterial load.

In conclusion, our study is the first to our knowledge demonstrating that the optimal antibiotic dose of a fluoroquinolone was markedly different between two bacterial species that exhibit similar MIC and are closely genetically related. The appropriate and prudent use of antimicrobials is a growing challenge for both human and veterinary medicine, and as already suggested, the optimization of antibiotic dosing regimens should take all its place in this challenge. In this context, our present results could stimulate further research to investigate the potential implementation of bacterial species-specific antibiotic doses in a clinical setting.
